# Mechanistic Explanations of Manual Therapy Do Not Influence Outcomes in Healthy Individuals: A Randomized Controlled Trial

**DOI:** 10.1002/hsr2.72467

**Published:** 2026-05-03

**Authors:** Sofie Waesch, Judith Leitner, Lisa M. Stelzer, Waclaw M. Adamczyk, Kerstin Luedtke, Tibor M. Szikszay

**Affiliations:** ^1^ Institute of Health Sciences, Department of Physiotherapy, Pain and Exercise Research Luebeck (P.E.R.L.) Universität zu Lübeck Lübeck Germany; ^2^ Center of Brain, Behavior and Metabolism (CBBM) Universität zu Lübeck Lübeck Germany; ^3^ Laboratory of Pain Research, Institute of Physiotherapy and Health Sciences, The Jerzy Kukuczka Academy of Physical Education Katowice Poland

**Keywords:** manual therapy, mechanisms, neurophysiological processes, pressure pain threshold, range of motion

## Abstract

**Background and Aims:**

Manual therapy is widely applied for musculoskeletal dysfunction and pain, yet the mechanisms underlying its effects remain incompletely understood. Local biomechanical and central neurophysiological processes are considered relevant, while contextual factors such as patient expectations and therapist beliefs may further influence treatment outcomes. This study investigated whether suggestions regarding the mechanisms of manual therapy modulate treatment effects on range of motion (ROM) and pressure pain threshold (PPT), both locally and at a remote site.

**Methods:**

Ninety‐three healthy participants were randomized, in a university campus laboratory, to one of three groups, each receiving a distinct mechanistic suggestion (biomechanical, neurophysiological, or none) prior to a standardized lumbar manual therapy intervention. The outcomes comprised lumbar ROM, PPT, and movement‐related pain, assessed before and after the intervention at the treated site and at a remote site, and analyzed using a general linear model (GLM).

**Results:**

All groups showed significant improvements in ROM over time (*p* = 0.01, *η*²_p_ = 0.07) and between assessment areas (*p* = 0.006, *η*²_p_ = 0.08). However, no main effect of group was detected (*p* = 0.40), and no significant two‐way or three‐way interactions emerged (all *p* > 0.05). Similarly, no between‐group differences were observed for PPT or movement‐related pain.

**Conclusion:**

Suggestions regarding the mechanisms of manual therapy did not influence treatment outcomes in healthy individuals. Cognitive modulation through mechanistic suggestions failed to alter ROM, PPT, or movement‐related pain.

## 1 Introduction

Manual therapy (MT) is among the most frequently employed interventions for musculoskeletal dysfunctions and pain [1], aiming to enhance range of motion, alleviate pain, and improve functional capacity [2, 3, 4]. Although widely used in acute and chronic low back pain, MT appears to confer only modest improvements in pain and function, with effects comparable to other conservative interventions [5, 6, 7].

Over the past two decades, a paradigm shift has redefined the conceptual framework of MT, moving beyond a purely biomechanical perspective [8, 9, 10]. While MT has been shown to influence segmental mobility [11, 12, 13], local muscle activity [14], and tissue structures, joints, and fascia [3], evidence suggests a neurophysiological model involving complex interactions within the central (spinal and supraspinal) and autonomic nervous systems [15]. Consistent with this hypothesis, MT‐induced short‐term effects have been observed in surrogate markers of autonomic activity, including alterations in skin conductance and heart rate variability [16, 17]. Functional imaging in animal models [18] and changes in the human withdrawal reflex [19] suggest involvement of spinal processes in observed MT responses. Moreover, studies have demonstrated MT‐associated cortical modulations, including changes in somatosensory‐evoked potentials [20, 21] and functional imaging findings implicating supraspinal pain‐modulation [22, 23, 24, 25, 26], which have been linked to activity of the descending pain inhibitory system [27].

Emerging evidence underscores the substantial influence of contextual factors on the efficacy of MT interventions [28, 29, 30]. These factors encompass both verbal and nonverbal communication by the therapist, the therapeutic setting, the patient–therapist relationship, a transparently discussed treatment approach, and patient expectations regarding the intervention [30]. However, the degree to which information can shape expectations remains insufficiently characterized; notably, a recent meta‐analysis questions whether contextual manipulations within therapeutic encounters can yield clinically meaningful benefits for patients with chronic musculoskeletal pain [31]. Nonetheless, a survey conducted by our research group among 569 physiotherapists revealed a striking heterogeneity in beliefs regarding the underlying mechanisms of MT [32]. Surprisingly, these beliefs accounted for a significant proportion of the therapist's perceived effectiveness of MT interventions. Indeed, patients' perceived efficacy of manual therapy is a strong predictor of treatment response [33]. However, it remains unclear to what extent patients' beliefs about the mechanisms underlying MT correspond to objectively measurable clinical outcomes. This study aimed to investigate, in an experimental setting involving healthy participants, whether a suggestive local biomechanical versus neurophysiological explanatory approach to the mechanisms of MT differentially influences post‐intervention pain sensitivity and mobility. The hypothesis posits that a biomechanical suggestive explanation affects only pressure sensitivity and mobility locally at the treated site, whereas a centrally suggestive explanation about neurophysiological processes extends its effects to distant regions, implicating a broader neurophysiological modulation of (pain) processing.

## 2 Methods

### 2.1 Study Design and Procedure

This study was designed as a randomized controlled trial with three intervention arms. A sample of healthy, pain‐free participants (*n* = 93) was randomly assigned to one of three groups, each receiving different suggestions regarding the underlying mechanisms of MT: local biomechanical suggestion, neurophysiological suggestion, or no suggestion. In all groups, both before and immediately after a lumbar mobilization technique, local and remote range of motion (ROM), pain at the end of movement, and pressure pain thresholds (PPT) were assessed. To ensure the effectiveness of the suggestion and to mask the true aim of the study, all participants were informed that the project aimed to compare mobilization techniques of varying intensities. Upon completion of the study, participants received comprehensive debriefing regarding the actual study objectives and the current evidence on MT mechanisms.

**Figure 1:**
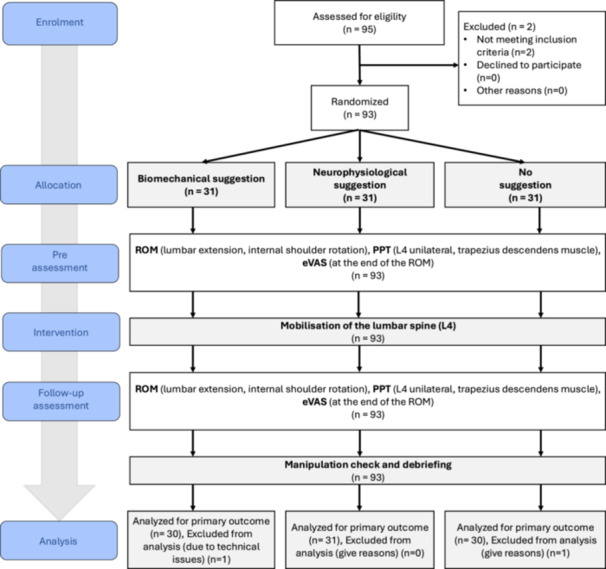
Schematic overview of the study flow and experimental procedure. Of 95 healthy individuals assessed for eligibility, 93 were randomized to one of three explanatory conditions: neurophysiological suggestion, biomechanical suggestion, or no suggestion. All participants underwent pre‐intervention assessment, lumbar spine mobilization at the L4 segment, and immediate post‐intervention assessment, followed by a manipulation check and debriefing. Outcome measures were obtained in randomized order and included lumbar extension range of motion (ROM), shoulder internal rotation ROM, end‐range pain intensity during the respective ROM task assessed using an electronic visual analog scale (eVAS), and pressure pain threshold (PPT) measured at the unilateral L4 level and at the descending trapezius muscle. Numbers in parentheses indicate the number of participants at each study stage.

Ethical approval was obtained from the Ethics Committee of the University of Lübeck prior to study initiation (Reference No. 2024‐103). The study protocol was preregistered on the Open Science Framework (https://osf.io/9hb3r). Although our experiment is a mechanistic trial without a clinical component, the entire study is reported in accordance with the Consolidated Standards of Reporting Trials (CONSORT) guidelines to ensure transparency and rigor. Data collection took place between January 2024 and May 2024 at the University of Lübeck. Participants were enrolled prior to randomization to ensure strict allocation concealment. The allocation to intervention arms as well as the order of measurements were determined using a generated randomization table created in Microsoft Excel (Microsoft Corporation, Version 16.108.1, Redmond, US) for each participant, which predefined both group assignment and the examination sequence. The randomization process (including assignment to the explanatory suggestions) was implemented by an independent individual who was not involved in any subsequent assessments or treatments, thereby minimizing the risk of selection and performance bias. Participants were blinded to the true study objective and remained unaware of the distinct explanatory suggestions provided. In addition, both outcome assessors (examiners) and treating personnel were blinded to group allocation with respect to the explanatory frameworks. All participants provided both oral and written informed consent before study participation and retained the right to withdraw from the study at any time without providing a reason. An overview of the study design is presented in Figure 1.

### 2.2 Participants

Healthy, pain‐free individuals between the ages of 18 and 65 years were eligible to participate if they subjectively reported being in good health and free of pain on the day of assessment. Exclusion criteria included a history of chronic pain lasting more than 3 months within the past 2 years, any painful episode lasting more than 30 min within the past week, pre‐existing shoulder or back pain, or a diagnosed neurological, cardiovascular, psychiatric, or systemic disorder. Participants were recruited from students and staff of the University Campus, as well as from their relatives and friends. Students and staff were approached via institutional email distribution lists, whereas recruitment beyond the university community was conducted using snowball sampling.

Given the lack of prior studies employing a comparable design to examine MT mechanisms in conjunction with cognitive manipulation and suggestion, the sample size was estimated using an effect size derived from a published meta‐analysis on the general impact of verbal suggestion on pain perception [34]. Specifically, the most conservative effect size reported (Cohen's *d* = 0.66) was used for the a priori power analysis. Assuming 80% power and a two‐sided significance level of *α* = 0.05, the analysis indicated that 30 participants per group (total *N* = 90) were required to detect a statistically significant difference between the experimental and control conditions, as calculated with the G*Power software [35].

### 2.3 Cover Story and Suggestion Intervention

Following written and verbal informed consent, participants viewed a standardized PowerPoint presentation (Version 16.93, Microsoft Corporation) accompanied by a scripted verbal narration, without exposure to an in‐person presenter. Each participant watched the generated video individually. The presentation provided uniform information on study procedures, measurement parameters, and the intervention. Participants were informed that the presentation ensured identical information for all groups. A study staff member monitored adherence during the presentation. The primary study objective, as outlined in a preceding study [36], was communicated to all subjects as an investigation of spinal mobilization effects on pain and range of motion, with a particular focus on the influence of different movement amplitudes.

As a part of the presentation, participants received additional information about the proposed mechanisms of spinal mobilization according to their group allocation: in the local biomechanical suggestion group, spinal mobilization was described as inducing local biomechanical effects that enhance joint mobility. These effects were presented as resulting from improved gliding of the facet joints under applied pressure, with subsequent changes in the stiffness of surrounding structures such as joint capsules, ligaments, myofascial tissues, and other connective tissues. The applied pressure was further described as directly influencing the tension of local paravertebral muscles, potentially leading to pain relief and increased local mobility. In the neurophysiological suggestion group, spinal mobilization was described as exerting systemic neurophysiological effects. These effects were framed as locally and remotely mediated processes contributing to pain modulation and mobility improvements. Evidence from basic science research was highlighted, suggesting that manual therapy activates brain regions associated with pain modulation and motor control, involving endogenous opioids and other neurotransmitters, which in turn may influence pain perception and mobility throughout the body. The control group received no additional mechanistic explanation.

### 2.4 Manual Mobilization

All included participants received a standardized manual posteroanterior (PA) mobilization technique targeting the lower lumbar spine, administered by a specially trained physiotherapist. This technique is commonly applied in both healthy individuals and patients with lower back pain [36, 37, 38, 39, 40]. During the mobilization, participants lay in a prone position on the treatment table with their hands placed under their foreheads. The PA mobilization was performed at the L4/L5 segment, identified using Tuffier's line as the midpoint between the iliac crests [41]. The intervention closely resembled a typical clinical session, consisting of three cycles of oscillatory PA pressure applied at a frequency of 0.5 Hz, corresponding to a Grade III mobilization—characterized by large‐amplitude movements, applied against resistance [42]. Each treatment cycle lasted 60 s and was followed by a 60‐second rest period.

The applied force during the Grade III mobilization was determined by the therapist (no verbal interaction occurred during the intervention), as substantial interindividual variability in segmental motion response to pressure has been reported [11, 13]. Contrary to the cover story provided to participants, the therapeutic effect of the mobilization was not defined by the applied pressure [36]. To maintain the appearance that pressure recordings were being conducted for control purposes, sham electrodes (standard self‐adhesive electrodes for transcutaneous electrical nerve stimulation) were affixed to the therapist's palm and connected to a computer. However, no pressure data were recorded, and no electrical stimulation was administered. Following the intervention, all participants were asked to rate the perceived average pain intensity of the mobilization on an electronic Visual Analog Scale (eVAS) ranging from 0 (no pain) to 100 (maximum pain).

### 2.5 Range of Motion

Range of motion (ROM) was assessed immediately before and after the intervention in all participants, including both evaluations of lumbar extension (local site) and shoulder internal rotation (remote site). To determine the active ROM of lumbar extension, participants were instructed to extend their lumbar spine maximally three times from a standing position (knees extended, feet hip‐width apart, arms crossed in front of the sternum), while their movement was recorded in a sagittal view using a 2D video camera (X‐T30, Fujifilm, Tokyo, Japan) [43]. High‐contrast markers were placed on the following anatomical landmarks: acromion, greater trochanter, and lateral femoral condyle. Motion analysis was conducted using Kinovea software (Version 0.8.15, Kinovea, 2023) to determine the angles between the acromion‐trochanter and trochanter‐lateral femoral condyle lines in the sagittal plane from the standing position to full extension [44]. Subsequently, participants rated the perceived pain intensity at the end of the motion on an eVAS (0 = no pain, 100 = maximum pain). The assessment of shoulder internal rotation ROM was performed with participants lying on their side on a treatment bench (hips and knees at 90° flexion). They were instructed to perform three maximal active internal rotations at 90° shoulder flexion with the elbow flexed at 90°. As with lumbar ROM, digital angle measurement (markers: olecranon, distal ulna) and subjective evaluation of movement quality (eVAS) were recorded. Each movement was demonstrated by the assessor once before the assessment, with corrections made if necessary.

### 2.6 Pressure Pain Thresholds

To evaluate pressure sensitivity, PPT were measured three times laterally to the spinous process of L4 (corresponding to the intervention area) and at the descending trapezius muscle (midway between the spinous process of C7/vertebra prominens and the acromion) [45, 46] using a digital pressure algometer (Medoc, Ramat Yishai, Israel). Standardized procedures for quantitative sensory testing, as outlined by the German Research Network on Neuropathic Pain (DFNS), were followed [47]. Participants were instructed to verbally indicate “stop” at the first sensation of pain, with pressure values (kg/cm²) recorded accordingly. The order of assessments (ROM, PPT) and the sequence of body regions examined (lumbar spine, shoulder region) were randomized.

### 2.7 Participant Characteristics

Data on participants' height, weight, sex (at birth), and dominant hand were collected. Additionally, participants were asked whether they had previously undergone spinal mobilization. The following questionnaires were administered to control for potential baseline differences: the Pain Vigilance and Awareness Questionnaire (PVAQ) [48], assessing attention to pain‐related stimuli; the Pain Sensitivity Questionnaire (PSQ) [49], estimating individual pain sensitivity; and the International Physical Activity Questionnaire (IPAQ) [50], evaluating physical activity levels.

### 2.8 Manipulation Check and Debriefing

To verify the effectiveness of the suggestion regarding the applied MT intervention, post‐hoc control questions were asked immediately after the final assessment. Namely, participants were asked to recall their impression immediately after the PowerPoint presentation and to indicate whether the suggested mechanisms had been convincing (yes or no). At the conclusion of the study, all participants were debriefed regarding the true study objective. Furthermore, they were provided with a summary of the current evidence on MT mechanisms, highlighting the complex interaction of neurophysiological and biomechanical mechanisms [15]. No adverse responses were reported due to the cover story and debriefing procedure.

### 2.9 Statistical Analysis

All statistical analyses were performed using IBM SPSS Statistics (Version 29, Armonk, NY, US). Descriptive statistics included absolute (*n*) and relative frequencies (%) according to variable characteristics. Continuous variables were summarized using means (*x̄*) and 95% confidence intervals (95%CI) or medians (with interquartile range, IQR) if non‐parametric. The normality of the outcome distributions was assessed using the Shapiro–Wilk test. One‐way analysis of variance (ANOVA) was performed for the variables age, weight (including F statistic, degrees of freedom (df), and *p* value). Height, sex, dominant hand, and previous experience were analyzed using a *χ*² test (*χ*² statistic, df). A Kruskal–Wallis test was performed for the PVAQ, PSQ, and IPAQ questionnaire (H statistic, df). The three repetitions of PPTs and ROM were averaged. A three‐way ANOVA was used to analyze the effects of ROM, eVAS, and PPT, with “group” (biomechanical suggestion, neurophysiological suggestion, no suggestion) as a between‐factor and “time” (baseline, post‐intervention) and “body area” assessed (local, distal) as within factors. If necessary, Bonferroni‐corrected post‐hoc *t*‐tests (two‐sided) were conducted if significant effects emerged. An exploratory Pearson correlation (*r*) analysis (two‐sided) was conducted to examine whether the individually perceived pain intensity during the intervention correlated with the outcome variables ROM, eVAS, and PPT (pre‐post difference). Furthermore, an exploratory data analysis also included a three‐way ANOVA to perform a subgroup analysis exclusively on female participants. The a priori level of significance was set at *p* < 0.05. The effect sizes for the ANOVAs are reported as partial eta squared (*η*_p_²), from Kruskal–Wallis tests as Kendall's W, and from *χ*² tests as Cramér's V.

## 3 Results

### 3.1 Characteristics

A total of 92 participants completed the study (local biomechanical suggestion: *n *= 30, neurophysiological suggestion: *n *= 31, no suggestion: *n *= 31). No side effects were reported as a result of MT. Apart from a significant difference between the groups regarding sex distribution, no significant differences in baseline characteristics were found between the groups (Table 1). Post hoc manipulation check revealed that all participants believed in the originally presented study objective. The Shapiro–Wilk test results for all primary outcomes are provided in the Supporting information S1: Table 1). Although the normality assumption was not met for all outcomes, groups and time points, parametric analyses were conducted, as the ANOVA is considered robust to moderate deviations from normality, particularly with approximately *n* = 30 participants per group [51].

**Table 1:** Baseline characteristics.

		Biomechanical suggestion (*n* = 30)	Neurophysiological suggestion (*n* = 31)	No suggestion (*n* = 31)	Statistics
Age	Years, *x̄* [95%CI]	25.5 [22.8, 28.1]	26.7 [22.9, 30.6]	24.7 [22.9, 26.6]	F(2, 91) = 0.52, *p *= 0.60, *η* ^2^_p _= 0.011^a^
BMI	cm, *x̄* [95%CI]	22.7 [21.4, 24.0]	23.1 [22.1, 24.1]	22.8 [21.7, 23.9]	F(2, 91) = 0.11, *p* = 0.90, *η* ^2^_p_ = 0.002^a^
Sex (at birth)	Female, *n* (%)	21 (70)	27 (87)	18 (58)	*χ* ^2^(2) = 6.5, *p *= 0.039, *V* = 0.27 b
Dominant hand	*n* (%)	28 (93)	29 (94)	29 (94)	*χ* ^2^(2) = 0.002, *p* = 0.99, V = 0.005 b
PVAQ	M (IQR)	35 (9.75)	40 (9)	37 (11.5)	H(2) = 3.2, *p* = 0.20, *W* = 0.04^c^
PSQ	M (IQR)	45 (25.75)	48 (23.5)	48 (27)	H(2) = 3.1, *p* = 0.22, W = 0.03^c^
IPAQ	Low *n* (%) Moderate *n* (%) High *n* (%)	0 (0) 8 (27) 21 (70)	0 (0) 7 (23) 24 (77)	1 (3) 11 (36) 19 (61)	H(2) = 2.2, *p* = 0.33, W = 0.03^c^
Previous MT experience	*n* (%)	6 (20)	4 (13)	9 (29)	*χ* ^2^(2) = 2.47, *p* = 0.29, *V* = 0.11 b

Abbreviations: 95%CI, 95% confidence interval; *η*_p_², partial eta squared; *x̄*, mean; BMI, body mass index; IPAQ, international physical activity questionnaire; IQR, interquartile range; M, Median; PSQ, pain sensitivity questionnaire; PVAQ, pain vigilance and awareness questionnaire; V, Cramér's V; W, Kendall's W.

^a^ one‐factorial analysis of variance.

^b^ *χ* ^2^ test.

^c^ Kruskal–Wallis test.

### 3.2 Range of Motion

**Figure 2:**
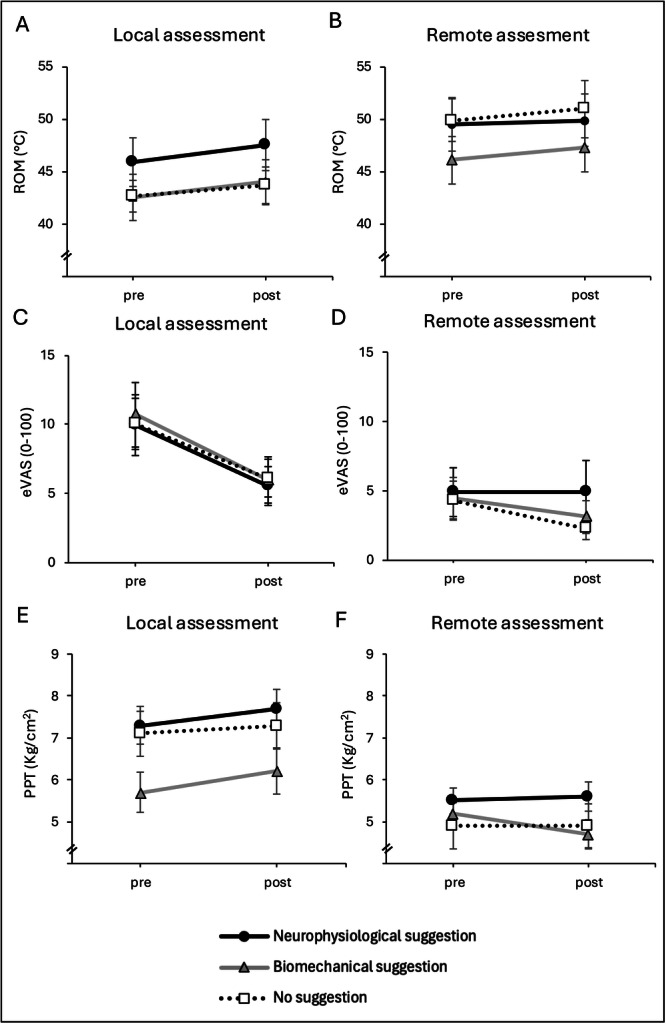
Effect of suggestive explanatory frameworks on range of motion, end‐range pain, and pressure pain threshold following manual therapy. Panels (A and B) show range of motion (ROM; (A) lumbar extension [local assessment]; (B) shoulder internal rotation [remote assessment]). Panels (C and D) show end‐range pain intensity during the corresponding ROM task, assessed using an electronic visual analog scale (eVAS; (C) lumbar extension; (D) shoulder internal rotation). Panels (E and F) show pressure pain threshold (PPT; (E) unilateral L4 [local assessment]; (F) descending trapezius muscle [remote assessment]). Local assessment refers to the lumbar region targeted by the manual therapy intervention, whereas remote assessment refers to an anatomically distant site. Measurements were obtained before (pre) and immediately after (post) the intervention. Neurophysiological suggestion is represented by the black solid line with circles, biomechanical suggestion by the grey solid line with triangles, and no suggestion by the dotted line with open squares. Data are presented as means ± standard error of the mean (SEM).

The ANOVA revealed significant main effects for the ROM assessment concerning the factors “time” (F(1,89) = 6.32, *p* = 0.01, *η* ^2^_p_ = 0.07) and “area”, (F(1,89) = 7.90, *p* = 0.006, *η* ^2^_p_ = 0.08). However, no significant main effect was observed for the factor “group,” (F(2,89) = 0.94, *p* = 0.40, *η* ^2^_p_ = 0.02). Furthermore, neither the two‐way interactions (“time” × “group”: (F(2,89) = 0.48, *p* = 0.95, *η* ^2^_p_ = 0.001; “area × “time”: (1,89) = 0.26, *p* = 0.61, *η* ^2^_p_ = 0.003; “group”×“area”: (2,89) = 0.68, *p* = 0.51, *η* ^2^_p_ = 0.02) nor the three‐way interaction (“time” × “area” × “group”: F(2,89) = 0.17, *p* = 0.84, *η* ^2^_p_= 0.004) were statistically significant. A detailed summary of the descriptive values is provided in Table 2 and Figure 2.

**Table 2:** Effects of different suggestions on range of motion, pain intensity, and pressure pain thresholds.

	ROM in °, *x̄* [95%CI]		eVAS 0–100, *x̄* [95%CI]		PPT kg/cm^2^, *x̄* [95%CI]	
Local assessment	Pre	Post	Pre	Post	Pre	Post
Neurophysiological suggestion	45.9 [41.2, 50.6]	47.5 [42.5, 53.5]	9.9 [5.4, 14.5]	5.5 [2.6, 8.3]	7.3 [6.4, 8.3]	7.7 [6.8, 8.6]
Biomechanical suggestion	42.5 [39.5, 45.6]	44.0 [40.3, 47.6]	10.7 [5.9, 15.5]	6.0 [2.6, 9.5]	5.7 [4.6, 6.8]	6.2 [5.1, 7.3]
No suggestion	42.7 [38.2, 47.1]	43.7 ([39.3, 48.1]	9.0 [5.2, 12.8]	6.1 [3.3, 8.9]	7.1 [6.1, 8.1]	7.3 [6.2, 8.4]
Remote assessment	Pre	Post	Pre	Post	Pre	Post
Neurophysiological suggestion	49.5 [44.3, 54.8]	49.9 [44.9, 55.0]	4.9 [1.3, 8.5]	4.9 [.3, 9.5]	5.5 [4.9, 6.1]	5.6 [4.9, 6.4]
Biomechanical suggestion	46.1 [42.0, 50.2]	47.3 [41.7, 52.9]	4.5 [1.5, 7.6]	3.1 [.8, 5.5]	5.2 [4.0, 6.4]	4.7 [3.6, 5.7]
No suggestion	49.9 [45.3, 54.6]	51.0 [46.3, 55.7]	4.3 [1.4, 7.3]	2.3 [.5, 4.0]	4.9 [4.1, 5.7]	4.9 [4.2, 5.7]
“time” × “area” × “group” interaction	F(2, 89) = 0.17,*p* = 0.84, *η* ^2^_p_ = 0.004		F(2, 89) = 1.14,*p* = 0.33, *η* ^2^_p_ = 0.03		F(2, 89) = 2.44,*p* = 0.09, *η* ^2^_p_ = 0.05	

*Note: x̄*: mean, 95%CI: 95% confidence interval, pre: before manual therapy (MT), post: directly after MT. Local assessment refers to measurements taken at the lumbar spine (Level L4), the area where MT was applied, while remote assessment includes measurements taken at sites distant from the MT application, specifically the trapezius descendens muscle for pressure pain thresholds (PPT) and shoulder internal rotation for range of motion (ROM). Pain intensity was evaluated using an electronic visual analog scale (eVAS, 0–100) at the end of ROM. Participants were divided into three groups based on the type of suggestion received: neurophysiologic (*n* = 31), biomechanic (*n* = 30), and no suggestion (*n* = 31). The triple interaction of the factors “time” × “area” × “group” from the analysis of variance is represented.

### 3.3 End of Range Pain Intensity

Regarding end of range pain intensity measured with the eVAS, significant main effects were observed for the factors “time” (F(1,89) = 13.11, *p* < 0.001, *η* ^2^_p_ = 0.13) and “area” (F(1,89) = 13.57, *p* < 0.001, *η* ^2^_p_ = 0.13), but not for “group” (F(2,89) = 0.14, *p* = 0.87, *η* ^2^_p_ = 0.003). Additionally, a significant two‐way “area” × “time” interaction was found (F(1,89) = 7.79, *p* = 0.006, *η* ^2^_p_ = 0.08), whereas no significant interactions were detected between “time” and “group” (F(2,89) = 0.11, *p* = 0.90, *η* ^2^_p_ = 0.002) or “group” × “area” (F(2,89) = 0.27, *p* = 0.77, *η* ^2^_p_ = 0.006). The “time” × “area” × “group” interaction was also not statistically significant (F(2,89) = 1.14, *p* = 0.33, *η* ^2^_p_ = 0.03).

### 3.4 Pressure Pain Thresholds

Pressure pain thresholds showed significant interactions for the factors “area” (F(1,89) = 117.79, *p* < 0.001, *η* ^2^_p_ = 0.57), “area” × “time” (F(1,89) = 7.66, *p* = 0.007, *η* ^2^_p_ = 0.08), and “area × group” (F(2,89) = 5.30, *p* = 0.007, *η* ^2^_p_ = 0.11). In contrast, no significant effects were found for the factors “time” (F(1,89) = 0.87, *p* = 0.35, *η* ^2^_p_ = 0.01), “group” (F(2,89) = 1.66, *p* = 0.20, *η* ^2^_p_ = 0.04), or their interaction (F(2,89) = 0.47, *p* = 0.63, *η* ^2^_p_ = 0.01). The “time” × “area” × “group” interaction was also not statistically significant (F(2,89) = 2.44, *p* = 0.09, *η* ^2^_p_ = 0.05).

### 3.5 Explorative Analysis

The three suggestion groups showed no significant difference in pain intensity ratings of the MT intervention measured with the eVAS (F(2,91) = 0.97, *p* = 0.39, *η*²_p_ = 0.02). Reported pain intensity in none of the three groups correlated significantly with the effect of the MT intervention (pre vs. post) for ROM, eVAS, or PPTs (*p* > 0.05, *r* < 0.3). Due to baseline differences in sex distribution, the influence of female participants was analyzed separately. The results showed no significant three‐way interactions for ROM, eVAS, or PPTs (ROM: F(2,63) = 0.13, *p* = 0.88, *η*²_p_ = 0.004; eVAS: F(2,63) = 0.49, *p* = 0.62, *η*²_p_ = 0.02; PPT: F(2,63) = 0.85, *p* = 0.40, *η*²_p_ = 0.03) in females only.

## 4 Discussion

This study investigated in healthy participants whether suggestive explanations of MT mechanisms—either local biomechanical or neurophysiological—differentially influence pressure pain sensitivity and mobility post‐intervention. It was hypothesized that a biomechanical explanation would affect mechanical sensitivity and mobility locally, whereas a neurophysiological explanation would induce broader effects, reflecting central modulation of pain processing. However, the results demonstrated no significant differences in ROM, PPT, or movement‐related pain across groups or assessed body regions, suggesting that the therapeutic effects of MT in healthy individuals are independent of suggestive contextual framing. These findings highlight the robustness of MT outcomes, indicating that explanatory suggestions do not alter its efficacy.

Overall, the evidence suggests that MT may improve spinal mobility and reduce pain, although observed benefits are generally short‐term and of small‐to‐moderate magnitude [6, 52, 53, 54, 55, 56]. For instance, Shum et al. [40] reported marked improvements in spinal kinematics and clinical pain following manual intervention in patients with back pain; notably, similar to the present study, these effects were assessed under laboratory conditions before and after the experimental session. As the resulting hypoalgesic effects were reported not to be confined to the treated region, Lascurain‐Aguirrebeña et al. proposed that MT elicits neurophysiological responses, including reduced mechanosensitivity and the induction of hypoalgesia [12]. Consequently, MT is expected to modulate the pressure‐induced pain systemically. Supporting this, Willett et al. observed that varying mobilization frequencies applied to the lumbar spine significantly influenced PPT in asymptomatic individuals [57]. Notably, they demonstrated that manual mobilization not only increased PPT but also elicited both localized and widespread hypoalgesic effects beyond the site of application. The available evidence suggests that vertebral‐level specificity of MT for nonspecific low back pain (LBP) is not associated with superior reductions in pain intensity [58], and that differences between MT approaches are generally small and likely not clinically meaningful, with most techniques yielding comparable pain relief [59]. Comparable results were observed in our study, with significant time interactions found for ROM, end‐range pain intensity, and PPT—likely attributable to the identical MT intervention applied across all groups. However, these effects were not confirmed in a recent systematic review and meta‐analysis conducted by our research group [60], which found no significant hypoalgesic effects for either local or remote pressure pain thresholds compared to the control group, thus failing to demonstrate effects beyond a simple pre‐post comparison. Similar ambiguities have been highlighted in a systematic review by Rodgers et al., which reported conflicting evidence concerning the hypoalgesic effects of MT as assessed through quantitative sensory testing [61]. Indeed, Keter et al., in their systematic review, describe MT as likely operating through a multifaceted interplay of biomechanical, neurophysiological, and other physiological processes [10]. They further underscore that the overall quality of the evidence is low to moderate, warranting cautious interpretation. Within this framework, the review supports peripheral, segmental‐spinal, and supraspinal mechanisms, while suggesting that the current evidence more strongly favors neurophysiological rather than purely biomechanical pathways. Moreover, the same authors highlight the growing relevance of contextual factors and other mediators of clinical response, emphasizing the need for more rigorous investigation of these variables in future research. In this context, patients' expectations and the influence of clinical setting factors have gained prominence [15, 28], particularly as they may contribute to the highly variable outcomes of MT.

Beyond the established biomechanical and neurophysiological mechanisms, a growing body of evidence suggests that patients' expectations can substantially shape therapeutic outcomes. Recent meta‐analysis showed moderate to large effects of verbal suggestion on pain reduction [62], emphasizing the critical role of expectations in modulating clinical responses. Expectations and cognitive framing may lead to placebo responses, which are further mediated by contextual variables within the therapeutic setting [15, 28]. Typically, such responses arise from verbal cues that align patients' expectations with anticipated treatment effects, a process heavily influenced by the therapist's communication style [28, 30]. Wilson et al. provided empirical evidence showing that positive expectations during massage therapy are related to greater hypoalgesic effects, whereas negative expectations either attenuated or even abolished these effects [63]. Similarly, Bialosky et al. demonstrated that post‐intervention pain following spinal MT was significantly modulated by pre‐intervention expectation framing, further substantiating the critical role of cognitive priming [64]. However, considering these findings, our study offers a nuanced contribution to this discourse. Although no significant between‐group differences in ROM, PPT, or movement‐evoked pain were observed, the objective was not to contrast positive versus negative expectations per se. Rather, this study investigated whether differing explanatory models—emphasizing either local biomechanical or neurophysiological mechanisms—could influence therapeutic outcomes. The absence of differential effects across our examined groups suggests that, within a healthy population, the outcomes of MT are robust to variations in suggestive explanatory context. This implies that in such populations, mechanistic framing may not meaningfully alter the efficacy of MT. Nevertheless, these findings are consistent with emerging perspectives that contextual and suggestive influences may be more pronounced in clinical populations, in which expectations, prior pain experience, and central sensitization may represent more salient mediators of treatment response [65, 66]. Importantly, however, even in musculoskeletal conditions, there may be limited potential to achieve clinically meaningful benefit for patients with chronic musculoskeletal pain solely by manipulating the contextual elements of non‐pharmacological and non‐surgical interventions [31].

General findings indicate that suggestive contextual framing does not alter ROM, pain, or mechanical sensitivity in healthy adults, suggesting that the efficacy of MT may be resilient to particular cognitive (mechanistic) suggestions. This stands in contrast to prior studies demonstrating that more positive or negative (emotionally) loaded suggestions can influence hypoalgesic responses [63], highlighting the potential limitations of neutral, mechanism‐focused framing alone. Further research is needed to investigate the effects of suggestion in symptomatic populations, as well as to explore alternative modes and timings of expectation delivery that may more effectively engage placebo‐related mechanisms in therapeutic contexts.

### 4.1 Limitations

This study employed a blinded randomized controlled design, validated outcome measures, and a standardized protocol for both the intervention and contextual framing, delivered via recorded presentations. Several limitations warrant consideration: First, randomization resulted in a baseline sex imbalance between groups; however, exploratory analyses suggested that this did not meaningfully confound the results. Second, the experiment was conducted under laboratory conditions in young, physically active, healthy participants, which may limit generalizability to symptomatic populations and routine clinical settings. Third, follow‐up was limited to the immediate post‐intervention assessment, thereby precluding conclusions about sustained effects; accordingly, the observed changes likely represent transient neuromodulatory responses to the brief sensory stimulus and may be confined to the period directly following the intervention rather than persisting thereafter. Fourth, although some outcomes reached statistical significance, the observed changes in pain and ROM remained below currently available thresholds for minimal clinically important difference or minimal detectable change reported in patient populations [5, 67, 68, 69]. Given that these thresholds originate from clinical samples, their applicability to healthy individuals is uncertain. However, this comparison provides useful context, suggesting that the magnitude of the observed effects is modest from a clinical perspective. Finally, it remains possible that participants' expectations influenced responses despite blinding procedures.

## 5 Conclusion

The present findings suggest that, in healthy individuals, outcomes following lumbar mobilization were not detectably modulated by the suggestive explanatory framing used. These interpretations should, however, be considered in light of therapist involvement in both treatment delivery and outcome assessment, as well as the possibility that participants' expectations may have influenced responses. Future research should test symptomatic populations, alternative suggestion timing and delivery, and clinically relevant settings to determine whether modified treatment‐related beliefs influence outcomes.

## Author Contributions

**Sofie Waesch:** conceptualization, data curation, formal analysis, investigation, methodology, writing – original draft, writing – review and editing. **Judith Leitner:** data curation, methodology, and writing – original draft. **Lisa M. Stelzer:** data curation, methodology, and writing – original draft. **Waclaw M. Adamczyk:** data curation, methodology, and writing – original draft. **Kerstin Luedtke:** conceptualization, formal analysis, investigation, methodology, project administration, resources, supervision, validation, writing – original draft, writing – review and editing. **Tibor M. Szikszay:** conceptualization, formal analysis, investigation, methodology, project administration, resources, supervision, validation, writing – original draft, writing – review and editing.

## Funding

The authors have nothing to report.

## Ethics Statement

Ethical approval was obtained from the Ethics Committee of the University of Lübeck prior to study initiation (Reference No. 2024‐103; January 8, 2024). This research adhered to the principles of the Declaration of Helsinki for research involving human participants and data.

## Consent

Informed consent was obtained from all participants in the included studies, ensuring their understanding of the study's purpose, procedures, and implications.

## Conflicts of Interest

The authors declare no conflicts of interest.

## Transparency Statement

The corresponding author, Tibor M. Szikszay, affirms that this manuscript is an honest, accurate, and transparent account of the study being reported; that no important aspects of the study have been omitted; and that any discrepancies from the study as planned (and, if relevant, registered) have been explained.

## Supporting information

Supporting File:

## Data Availability

All data and materials utilized in this study are accessible to researchers upon request through an official academic email address.
