# Supplementary material for: Mechanistic Explanations of Manual Therapy Do Not Influence Outcomes in Healthy Individuals: A Randomized Controlled Trial
Source: Health Sci Rep. 2026 May 3;9(5):e72467. doi: 10.1002/hsr2.72467 (PMC13136611; doi:10.1002/hsr2.72467)
Supplement: Supplementary file 1 — Supporting File: [file HSR2-9-e72467-s001.docx]

**Suppinfo Tabe 1. Normality assessment of primary outcomes using the Shapiro–Wilk test**

| **Assessment** | | | **Group** | **Shapiro-Wilk** | | |
| --- | --- | --- | --- | --- | --- | --- |
| **Time** | **Outcome** | **Area** |  | **Statistic** | **df** | **Sig.** |
| Pre | PPT | local | Neurophysiological suggestion | 0.981 | 31 | 0.840 |
|  |  |  | Biomechanical suggestion | 0.800 | 30 | **<0.001** |
|  |  |  | No suggestion | 0.960 | 31 | 0.290 |
|  |  | remote | Neurophysiological suggestion | 0.970 | 31 | 0.517 |
|  |  |  | Biomechanical suggestion | 0.811 | 30 | **<0.001** |
|  |  |  | No suggestion | 0.916 | 31 | **0.019** |
|  | ROM | local | Neurophysiological suggestion | 0.976 | 31 | 0.682 |
|  |  |  | Biomechanical suggestion | 0.965 | 30 | 0.405 |
|  |  |  | No suggestion | 0.973 | 31 | 0.606 |
|  |  | remote | Neurophysiological suggestion | 0.961 | 31 | 0.314 |
|  |  |  | Biomechanical suggestion | 0.949 | 30 | 0.161 |
|  |  |  | No suggestion | 0.978 | 31 | 0.763 |
| post | PPT | local | Neurophysiological suggestion | 0.976 | 31 | 0.688 |
|  |  |  | Biomechanical suggestion | 0.880 | 30 | **0.003** |
|  |  |  | No suggestion | 0.936 | 31 | 0.065 |
|  |  | remote | Neurophysiological suggestion | 0.964 | 31 | 0.365 |
|  |  |  | Biomechanical suggestion | 0.712 | 30 | **<0.001** |
|  |  |  | No suggestion | 0.953 | 31 | 0.191 |
|  | ROM | local | Neurophysiological suggestion | 0.973 | 31 | 0.613 |
|  |  |  | Biomechanical suggestion | 0.967 | 30 | 0.451 |
|  |  |  | No suggestion | 0.961 | 31 | 0.302 |
|  |  | remote | Neurophysiological suggestion | 0.975 | 31 | 0.662 |
|  |  |  | 2 | 0.971 | 30 | 0.568 |
|  |  |  | No suggestion | 0.972 | 31 | 0.565 |
| pre | eVAS | local | Neurophysiological suggestion | 0.778 | 31 | **<0.001** |
|  |  |  | Biomechanical suggestion | 0.805 | 30 | **<0.001** |
|  |  |  | No suggestion | 0.814 | 31 | **<0.001** |
|  |  | remote | Neurophysiological suggestion | 0.533 | 31 | **<0.001** |
|  |  |  | Biomechanical suggestion | 0.607 | 30 | **<0.001** |
|  |  |  | No suggestion | 0.613 | 31 | **<0.001** |
| post | eVAS | local | Neurophysiological suggestion | 0.712 | 31 | **<0.001** |
|  |  |  | Biomechanical suggestion | 0.674 | 30 | **<0.001** |
|  |  |  | No suggestion | 0.765 | 31 | **<0.001** |
|  |  | local | Neurophysiological suggestion | 0.426 | 31 | **<0.001** |
|  |  |  | Biomechanical suggestion | 0.559 | 30 | **<0.001** |
|  |  |  | No suggestion | 0.562 | 31 | **<0.001** |

Pre: before manual therapy (MT); post: directly after MT. Local assessment refers to measurements taken at the lumbar spine (Level L4), the area where MT was applied, while remote assessment includes measurements taken at sites distant from the MT application, specifically the trapezius descendens muscle for pressure pain thresholds (PPT) and shoulder internal rotation for range of motion (ROM). Pain intensity was evaluated using an electronic visual analog scale (eVAS, 0–100) at the end of ROM. Participants were divided into three groups based on the type of suggestion received: Neurophysiologic (n = 31), Local Biomechanic (n = 30), and No Suggestion (n = 31) (df, degrees of freedom; sig, significance).
